# Prevalence of Psoriatic Arthritis in Patients with Moderate-to-Severe Psoriasis in the Era of Biologics and Small Molecule Therapies

**DOI:** 10.3390/jcm14238359

**Published:** 2025-11-25

**Authors:** Cristina Vergara-Dangond, Tatiana Cobo-Ibáñez, Gabriela Cueva-Nájera, Ricardo Valverde-Garrido, Cristina García-Yubero, Laura Trives-Folguera, Beatriz Paredes-Romero, Ana Victoria Esteban-Vázquez, Liz Romero-Bogado, Isabel De La Cámara-Fernández, Martina Steiner, Patricia Richi-Alberti, Ana Valeria Acosta-Alfaro, Iolanda Prats, Santiago Muñoz-Fernández

**Affiliations:** 1 Department of Rheumatology, Hospital Universitario Infanta Sofía, 28702 Madrid, Spain; gabrielanataly.cueva@salud.madrid.org (G.C.-N.); laura.trives@salud.madrid.org (L.T.-F.); mbeatriz.paredes@salud.madrid.org (B.P.-R.); anavictoria.esteban@salud.madrid.org (A.V.E.-V.); mromerob@salud.madrid.org (L.R.-B.); isabeldela.camara@salud.madrid.org (I.D.L.C.-F.); martina.steiner@salud.madrid.org (M.S.); patricia.richi@salud.madrid.org (P.R.-A.); anavaleria.acosta@salud.madrid.org (A.V.A.-A.); 2 Department of Medicine, Faculty of Medicine, Health and Sports, Universidad Europea de Madrid, 28670 Madrid, Spain; ricardo.valverdega@salud.madrid.org (R.V.-G.); cristina.gyubero@salud.madrid.org (C.G.-Y.); iolanda.prats@salud.madrid.org (I.P.); 3 FIIB HUIS-HUHEN, 28702 Madrid, Spain; 4 Department of Dermatology, Hospital Universitario Infanta Sofía, 28702 Madrid, Spain; 5 Department of Pharmacy, Hospital Universitario Infanta Sofía, 28702 Madrid, Spain

**Keywords:** prevalence, psoriasis, psoriatic arthritis, biologics

## Abstract

**Objectives:** To estimate the prevalence of psoriatic arthritis (PsA) and associated factors in patients with moderate-to-severe psoriasis. **Methods:** Retrospective, single-center study of a cohort of psoriasis patients in standard follow-up in a dermatology department from July 2008 to January 2024. Patients ≥18 years with moderate-to-severe psoriasis were included and classified into three groups according to the treatment received: group 1, biologics or small molecules with or without conventional synthetic disease-modifying anti-rheumatic drugs (csDMARDs); group 2, only csDMARDS; and group 3, non-pharmacological treatments. Demographic and clinical variables were collected. The prevalence of PsA was estimated with its 95% confidence interval (CI). The cumulative incidence of PsA was analyzed across groups, and logistic regression models were built. **Results:** The study population comprised 308 patients (67.2%, 22.7%, 10% in groups 1, 2, and 3, respectively). Differences between the groups were observed in severity of psoriasis, weight, smoking status, and dyslipidemia (*p* < 0.05). The prevalence of PsA was 11.7% (95% CI, 8.1–15.3), with most patients in group 1. This group had a higher risk of PsA following diagnosis of psoriasis or initiation of treatment. Belonging to groups 2 and 3 had a smaller effect than belonging to group 1 in the development of PsA; nail involvement and obstructive sleep apnea (OSA) were associated with development of PsA (*p* < 0.05). **Conclusions:** The prevalence estimate was lower than previous estimates, probably owing to the increased use of biologics. Nail involvement and OSA were associated with PsA.

## 1. Introduction

Psoriasis is an immune-mediated inflammatory disease (IMID) affecting the skin, nails, and scalp. It is present in 2.3% of the Spanish population [1]. Psoriatic arthritis (PsA) is a highly heterogeneous IMID in terms of its presentation, which comprises psoriasis, uveitis, inflammatory bowel disease, dactylitis, enthesitis, peripheral arthritis, and axial involvement [2]. Its high systemic inflammatory burden can lead to comorbidities such as cardiometabolic diseases. Furthermore, it can affect mental health, leading to anxiety and depression, which together pose a challenge to its management [3,4,5,6,7]. Up to 30% of patients with psoriasis develop PsA [8,9,10]. PsA usually follows the onset of psoriasis, although around 10% of patients develop skin manifestations after the onset of psoriatic arthritis [11].

Various models have been proposed to explain the transition from psoriasis to PsA [12], and it is known that both diseases share pathogenic mechanisms involving inflammatory cytokines [13,14]. Therefore, it is now proposed that both IMIDs be considered psoriatic disease. In this context, efficient control of psoriasis or even remission could reduce the risk of PsA. Although it is not known which treatments induce this preventive effect, it makes sense to target common pathways in the pathogenesis of psoriatic disease using agents such as TNF inhibitors, anti-interleukin agents (IL-12/23, IL-23, and IL-17), phosphodiesterase inhibitors, and Janus kinase inhibitors [15,16].

Several studies have investigated the hypothesis that treating moderate-to-severe psoriasis prevents PsA. A retrospective study by Gisondi et al. [17] demonstrated a lower incidence of PsA in patients with psoriasis undergoing biological therapy than in those undergoing phototherapy. Rosenthal et al. [18] also identified a lower risk of developing PsA among patients treated with biologic therapy versus conventional synthetic disease-modifying anti-rheumatic drugs (csDMARDs), whether or not they were combined with phototherapy. The PAMPA study is presently underway [19]. This multicenter, randomized, double-blind, placebo-controlled trial is comparing the transition to PsA in patients with psoriasis at an increased risk of progression who are treated with guselkumab versus standard non-biologic therapy.

Therefore, we believe that determining the prevalence of PsA in patients with psoriasis treated with currently available therapies (biologics, targeted therapies, csDMARDs, and non-pharmacological treatments) is an initial step that could aid in the early diagnosis of PsA and potentially reduce the transition from psoriasis to PsA. The aim of our study was to determine the prevalence of PsA in patients with moderate-to-severe psoriasis and analyze whether there are differences between the various standard treatments used for psoriasis. Furthermore, we aimed to identify factors associated with the development of PsA.

## 2. Materials and Methods

### 2.1. Study Design and Setting

This is a retrospective, observational, longitudinal study of a cohort of patients with moderate-to-severe psoriasis in standard follow-up in the dermatology department at Hospital Universitario Infanta Sofía, San Sebastián de los Reyes, Madrid, Spain, from July 2008 to January 2024.

### 2.2. Population

The study population comprised patients ≥18 years diagnosed with moderate-to-severe psoriasis who initiated standard treatment. Moderate-to-severe psoriasis was defined as a Psoriasis Area and Severity Index (PASI) score ≥ 6 and according to dermatological criteria, including psoriasis at selected sites such as the scalp, face, genitalia, nails or palms, and soles. Patients were classified according to the treatment received during the study period, as follows: group 1, who received biologics or small molecules with or without csDMARDs; group 2, who received only csDMARDs; and group 3, who received non-pharmacological treatments such as phototherapy or topical treatment. Patients with metabolic and inflammatory arthropathies or autoimmune diseases were excluded.

### 2.3. Data Collection

A rheumatologist with more than 10 years’ experience in diagnosing and treating patients with PsA extracted pseudo-anonymized data from medical records from the diagnosis of psoriasis until the diagnosis of PsA or the end of the study in January 2024. A clinical research associate was responsible for the external monitoring of patient data.

### 2.4. Variables

The main study variable was the development of PsA, which was diagnosed based on the CASPAR criteria [20]. To estimate the risk of developing PsA, patient follow-ups were defined, as follows: (1) the period between the date of diagnosis of psoriasis and that of the diagnosis of PsA or the end of the study in January 2024; and (2) the period between the date of initiation of treatment for moderate-to-severe psoriasis and the date of diagnosis of PsA or the end of the study in January 2024.

Secondary variables included the following: sociodemographic data, such as age and sex; variables related to psoriasis, such as type of psoriasis, severity of psoriasis (measured by PASI) [21], family history of psoriasis, time from onset of symptoms of psoriasis to diagnosis, time from diagnosis of psoriasis to treatment; variables related to PsA such as type of PsA and family history of PsA; time from onset of psoriasis symptoms to diagnosis of PsA; time from diagnosis of psoriasis and diagnosis of PsA; time from treatment of psoriasis and diagnosis of PsA; time from onset of musculoskeletal symptoms and diagnosis of PsA; comorbidities such as overweight/obesity, hypertension, hyperuricemia, dyslipidemia, diabetes, smoking, alcohol consumption, chronic obstructive pulmonary disease, obstructive sleep apnea (OSA), hepatic steatosis, and depression; extracutaneous variables such as musculoskeletal symptoms (joint pain, joint effusion, dactylitis, enthesitis, or inflammatory back pain), uveitis, inflammatory bowel disease; C-reactive protein (CRP) levels; HLAB27 positivity; and types of treatments.

### 2.5. Study Size

Considering that 23.8% of patients with psoriasis will also develop PsA [22], a 95% level of confidence, and a 5% precision error, it was estimated that at least 307 patients would be needed.

### 2.6. Statistical Analysis

Categorical variables were expressed as absolute (n) and relative frequencies (%), and 95% confidence intervals for proportions were calculated where appropriate. Continuous variables were expressed as mean ± standard deviation (SD) or median and interquartile range (IQR: p25–p75), depending on the distribution of the data, as assessed by the Kolgomorov–Smirnov test. The three treatment groups were compared using the chi-square test or Fisher’s exact test for categorical variables, as appropriate. For continuous variables, 1-way ANOVA was used when the data followed a normal distribution, and the Kruskal–Wallis test was applied for non-normally distributed variables. Post hoc analyses with a Bonferroni correction were conducted to adjust for multiple comparisons.

A logistic regression analysis was performed to identify independent factors associated with the onset of PsA. Variables that showed a statistically significant association in the univariate analysis (*p* < 0.100) were included in the multivariate model. Kaplan–Meier survival curves were generated to visualize the cumulative incidence of PsA across the groups, and differences between curves were assessed using the log-rank test.

All statistical analyses were performed using IBM SPSS Statistics, version 27 (IBM Corp., Armonk, NY, USA). A *p*-value < 0.05 was considered statistically significant.

### 2.7. Ethical Considerations

This study was conducted in accordance with the principles of the Declaration of Helsinki [23] and approved by the Clinical Research Ethics Committee of Hospital Universitario de Getafe, Getafe (Madrid), Spain (study code: A04/24) on 25 April 2024. The need for informed consent was waived because of the retrospective nature of the study.

## 3. Results

### 3.1. Sociodemographic Characteristics of Patients with Moderate-to-Severe Psoriasis

A total of 308 patients with moderate-to-severe psoriasis were included. Mean age was 55.7 ± 14.7 years, and 49.4% were women. Patients were distributed as follows: 207 (67.2%), 70 (22.7%), and 31 (10%) in groups 1, 2, and 3, respectively. Median time from diagnosis of psoriasis until the first treatment was 9 (2–19), 4 (0–15), and 0 (0–0) years in groups 1, 2 and 3, respectively. Differences between groups were observed for severity of psoriasis, being a smoker or former smoker, being overweight or obese, and dyslipidemia, with higher values recorded in group 1. As expected, patients in group 1 received a greater number of treatments than the other groups, except for phototherapy (Table 1).

In group 1, 107 (34.7%), 70 (22.7%), 21 (6.8%), 7 (2.3%), and 2 (0.6%) patients received 1, 2, 3, 4, or 5 biologics or small molecules, respectively.

### 3.2. Prevalence of PsA

A total of 36 patients were diagnosed with PsA. The general prevalence of PsA was 11.7% [95% CI, 8.1–15.3]. Prevalence according to groups was 15.5% [95% CI, 10.5–20.4], 4.3% [95% CI, 0.0–9.0], and 3.2% [95% CI, 0.0–9.5] in groups 1, 2, and 3, respectively.

### 3.3. Characteristics of PsA Patients

The mean age was 55.7 ± 15.5 years, and 52.8% were women.

The most frequent type of psoriasis was plaque psoriasis, with a mean PASI score of 8. The median time from onset of psoriasis symptoms to diagnosis of psoriasis and PsA was 7 months and 11 years, respectively. Peripheral PsA was the most frequent type. In addition, 38.9% and 13.9% of the patients had a family history of psoriasis and PsA, respectively. Regarding comorbidities, the most frequent were smoking or former smoking, overweight or obesity, dyslipidemia, and hypertension. OSA was recorded in 13.9%. As for extracutaneous variables, 1 (2.8%) and 6 (17.6%) patients had uveitis or were HLA-B27-positive, respectively (Table 2). The distribution of treatments that the 36 patients with psoriasis were receiving at the time they were diagnosed with PsA was as follows: csDMARDs, 16 (44.4%); biologics, 11 (30.6%); and non-pharmacological treatments, 9 (25%).

Of the 36 patients, 32 were in group 1, 3 in group 2, and 1 in group 3. Time from diagnosis of psoriasis, first treatment, and presence of musculoskeletal symptoms until diagnosis of PsA was longer in group 2. Median time from diagnosis of psoriasis until the first treatment was started in groups 1, 2, and 3 was 10.5 [2–19], 2.5 [1–17], and 0 [0–0] years, respectively (Table 2).

As for therapy, the most used csDMARD was methotrexate (83.3%), and all patients received phototherapy. Specifically in group 1, the most used biologic or small molecule was TNF inhibitors (63.9%), followed by anti–IL12/23 agents (33.3%), anti–IL-23 agents (30.6%), and apremilast (30.6%). In this group, 11 (34.4%), 14 (43.7%), and 4 (12.5%) patients received 1, 2, or 3 biologic or small molecules, respectively (Table 2).

### 3.4. Risk of Developing PsA

**Figure 1:**
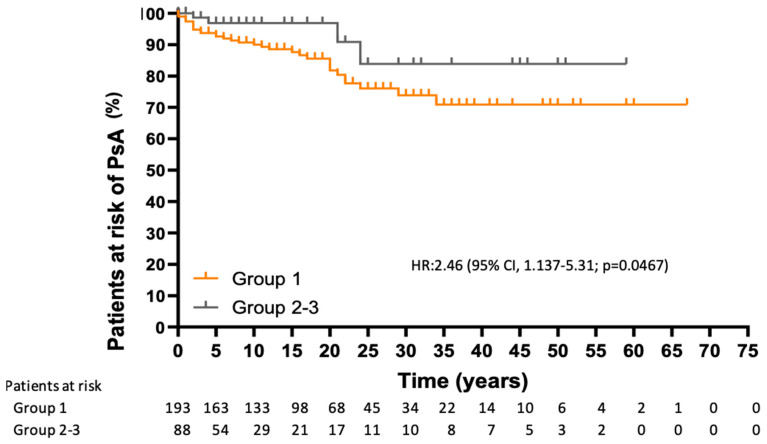
Time from diagnosis of psoriasis to diagnosis of PsA. HR: hazard ratio; CI: confidence interval.

**Figure 2:**
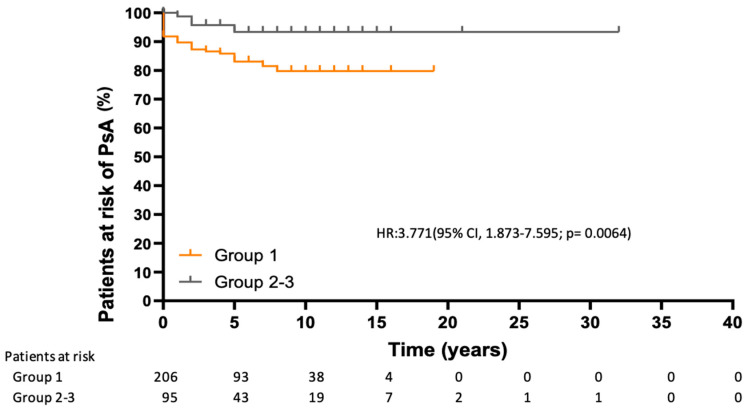
Time from initiation of treatment until diagnosis of PsA. HR: hazard ratio; CI: confidence interval.

Analysis of the risk of developing PsA over time revealed statistically significant differences, with a higher risk in group 1 considering the time from the diagnosis of psoriasis (HR, 2.46 [95% CI, 1.137–5.31]; *p*= 0.0467) and from initiation of psoriasis treatment (HR, 3.771 [95% CI, 1.873–7.595]; *p*= 0.006) (Figure 1 and Figure 2).

### 3.5. Factors Related to the Development of PsA

Table 3 shows the results of the univariate and multivariate analyses.

According to the multivariate model, the variables that were significantly associated with onset of PsA comprised OSA (OR, 19.498 [95% CI, 3.492–108.868]; *p* < 0.001), treatment group (OR, 0.262 [95% CI, 0.076–0.905], *p* = 0.034 for group 2 and OR, 0.067 [95% CI, 0.006–0.755], *p* = 0.029 for group 3), and nail involvement (nail only or nail and skin) (OR, 3.605 [95% CI, 1.660–7.830]; *p* = 0.001).

## 4. Discussion

This observational, retrospective study included 308 patients with moderate-to-severe psoriasis, of whom 207, 70, and 31 belonged to groups that received biologics or small molecules (with or without csDMARDs), only csDMARDs, or no pharmacological treatment, respectively, during the study period. We found clinical differences between these treatment groups. The overall prevalence of PsA was 11.69% (95% CI, 8.10–15.28), with most patients in the group receiving biologics or small molecules. This group also had a higher risk of PsA following diagnosis of psoriasis or initiation of treatment. The csDMARDs and non-pharmacological treatment groups had a smaller effect on the development of PsA than the biologics or small molecules group, while nail involvement and OSA were associated with the development of PsA.

The prevalence of PsA among patients with psoriasis is variable and is influenced by multiple factors. Two recent meta-analyses have examined the global and regional prevalence of PsA in patients with psoriasis.

Alinaghi et al. [22] collected the results of 266 studies from around the world published up to 2017, estimating the overall pooled prevalence to be 19.7% (95% CI, 18.5–20.9%). This result varied according to geographical distribution from 35.5% (95% CI, 11.8–64.0%) in Thailand to 4.9% (95% CI, 1.9–9.3%) in China. In Spain, the pooled prevalence was 18.7% (95% CI, 15.0–22.7%). In addition to differences in geographical distribution, the authors found that heterogeneity in the estimates was caused by differences in methodology, including the method used to define PsA, year of publication, disease severity, and age at diagnosis (adults), study design, and population size. A subsequent meta-analysis of 346 studies published up to 2023 showed that after adjusting for the covariates of heterogeneity, the global prevalence of PsA in patients with psoriasis was estimated to be 17.58% (95% CI, 3.33–43.69%), with a prevalence in Spain of 13.93 (95% CI, 9.67–19.33). As in the previous meta-analysis, the authors found that the heterogeneity in the estimates was affected by the same factors [24]. Our prevalence of 11.69% (95% CI, 8.10–15.28) fell within the range of the estimate made in the meta-analysis by Kang et al. [24]. The main novelty of our study is that the prevalence of PsA was obtained from a population of adult patients with moderate-to-severe psoriasis, all of whom were undergoing treatment. Another factor that may influence the prevalence value is the effect of psoriasis treatment (e.g., early biological therapies) on the development of PsA [25]. Many prevalence studies do not specify whether patients were receiving treatment for psoriasis or the type of treatment, nor do they analyze prevalence according to the treatment received [22,24,26]. In our sample, most patients (two-thirds) were receiving biologics or small molecules during the study period, reflecting current management according to clinical guidelines for patients with moderate-to-severe psoriasis [27,28]. This could account for our prevalence being lower than that observed in studies from earlier periods, in which treatment was not introduced as early and biologics were not as widely used. Furthermore, it would support the hypothesis that biological therapy for psoriasis could prevent the development of PsA.

In this regard, studies conducted in recent years investigated the preventive capacity of biological treatment in the development of PsA. Although most would support this hypothesis, contradictory results have been reported. Gisondi et al. [17] demonstrated a lower incidence of PsA in patients with psoriasis undergoing biological therapy than in those undergoing phototherapy. Rosenthal et al. [18] also identified a lower risk of developing PsA with biologics than with csDMARDs. Watad et al. [29] showed that biological agents are more effective than methotrexate in reducing incident PsA. By comparing with patients who were never treated with biologics, Floris et al. [25] found that different classes of biologics reduce the likelihood of peripheral and axial PsA, without distinguishing between different types of topical and systemic treatments. However, Acosta-Felquer et al. [30] identified a lower risk of PsA in patients treated with bDMARDs compared to topical treatments but not compared to csDMARDs. In addition, Meer et al. [31] found that the use of biologic therapies was associated with the development of PsA in patients with psoriasis. These studies are retrospective and heterogeneous in terms of design (observational [17,25,30] and population-based [18,29,31]), follow-up time, the context in which psoriasis and PsA were diagnosed, the types of population evaluated, and the criteria used for diagnosis. For this reason, clinical trials have been initiated to obtain a higher level of evidence to support this preventive effect [18].

Of the 36 patients diagnosed with PsA in our study, only 30% were taking biologics at the time of diagnosis; the remainder were taking csDMARDs (44.4%) and non-pharmacological treatment (25%). However, 32/36 patients who developed PsA belonged to the biologics and small molecules group, because they received these treatments for control of psoriasis throughout the study period. In this regard, the survival curves in our study showed that patients receiving biologics and small molecules were at greater risk of developing PsA than the other treatment groups. This increased risk was not only present from the start of treatment, but also from the diagnosis of psoriasis, thus supporting the idea that the development of PsA is determined by other factors from the onset of psoriasis or prior to it [8]. We found that patients receiving biologics and small molecules had more pronounced disease activity (PASI), a higher number of systemic treatments (≥2 biologics, 48%, with csDMARDs in at least 75%) and were more frequently obese, potentially indicating an additive effect and accounting for the observation that belonging to the csDMARD and non-pharmacological treatment groups has a smaller effect on the development of PsA than belonging to the biologics and small molecules group. Although the PASI score was not associated with the development of PsA, its predictive value has been shown to be weak and to increase when severity is defined by the need for systemic treatments [8]. Therefore, the group of biologics and small molecules in our study is the one with the most severe psoriasis. In addition, our study population already had an increased risk of developing PsA owing to moderate-to-severe activity of psoriasis, and on this basis, we detected, as have other authors, an association between PsA and nail involvement [8]. Similarly, musculoskeletal symptoms were associated with the development of PsA, since 100% of patients with PsA previously had these symptoms. On the other hand, there is no direct evidence that OSA is associated with PsA in patients with psoriasis. We found a limited number of studies identifying OSA as a factor associated with the development of psoriasis [32,33]. We even observed a bidirectional association, as both processes share a proinflammatory state coinciding with inflammatory pathways and cytokines [34]. Given that both psoriasis and PsA constitute psoriatic disease, OSA could also be related to the development of PsA in affected patients. This finding from our study is interesting, although it must be confirmed in further research.

The main strength of this study is that it reflects daily clinical practice in the management of patients with moderate-to-severe psoriasis. The exponential increase in the use of biologics in recent years in this patient population is reflected in a lower prevalence of PsA than previous estimates in Spain [22,26]. The observational design does not present coding or classification problems and allows for the inclusion of clinical and demographic variables. Consequently, our design differs from that of other studies that are epidemiological and collect variables from registries not originally designed for research purposes [18,29,31]. Furthermore, we believe it is appropriate to investigate a population that is at higher risk of PsA because of disease activity. In turn, the use of a validated index such as the PASI allows for a more precise definition of the population with moderate-or-severe activity than using, as in other studies, the type of prescription (biological treatments vs. non-biological treatments, systemic treatments vs. non-systemic treatments) [8].

Our study is subject to limitations. First, the accuracy of the data may be affected by the retrospective design. Specifically, the obesity/overweight variable may be underestimated, since body mass index was not recorded for all patients in the dermatology outpatient clinic, thus explaining why it was not associated with PsA in our multivariate analysis. This could be an important limitation of this study. However, the accuracy of the data was improved by using external monitoring to identify errors and missing data, which were corrected and completed, if necessary, by telephoning the patients or contacting general practitioners. Second, PsA screening tools such as PURE-4 (Psoriatic arthritis UnclutteRed screening Evaluation) test are used in the psoriasis outpatient clinic, and a rheumatologist is consulted on the same day to identify patients in whom the presence of PsA should be investigated if the PURE test is ≥1. Third, a possible confounding bias is that a patient may have received multiple treatments of varying duration, making it difficult to relate the development of PsA to a single treatment. Four, although we have evaluated in multivariable models the duration of psoriatic disease, the time from the onset of symptoms to the diagnosis of psoriatic arthritis and the start of psoriasis treatment, we have not recorded the time of the patient on each treatment group, which may bias some results. Finally, participants were distributed unevenly between treatment groups, with a predominance of group 1. The cohort analyzed is representative of the Spanish population, and the clinical setting closely reflects real-world scenarios. Generalization of the results might be restricted to healthcare settings with similar patient demographics, prescribing behaviors, and treatment availability as in Spain’s psoriasis population.

In our study, patients receiving biologics or small molecules likely had more severe or long-standing psoriasis, which could increase the risk of developing psoriatic arthritis. Therefore, the observed higher PsA risk in the biologic group may reflect disease severity rather than a true treatment effect. Based on our study, it cannot be inferred that patients treated with biologics or small molecules have a real increase in PsA. The fact that these groups typically include the most severely ill patients may, as a confounding factor, lead to PsA being more frequent compared to the other group, and therefore, causal inferences cannot be drawn from the present design. To overcome our limitations and those of other retrospective studies, we plan to develop a prospective multicenter study to determine the incidence of PsA among patients with psoriasis.

## 5. Conclusions

In conclusion, our retrospective study of patients with moderate-to-severe psoriasis revealed a predominance of biologics over other treatments, and in this context, a lower prevalence of PsA than estimates made in years prior to the study period. Nail involvement is well known to be associated with PsA. A novel and interesting finding of the present study was the association between OSA and PsA.

## Figures and Tables

**Table 1:** Demographic and clinical characteristics of psoriasis patients.

	Total n = 308	Group 1 n = 207 (67.2%)	Group 2 n = 70 (22.7%)	Group 3 n = 31 (10.1%)	*p* Value
Demographic data					
Age	55.7 ± 14.75	56.5 ± 14.4	55.9 ± 16.1	49.8 ± 13.1	NS
Female sex	152 (49.4)	100 (48.3)	37 (52.9)	15 (48.4)	NS
Variables related to psoriasis					
Type of psoriasis					
Plaque	141(45.8)	79 (38.2)	42 (60)	20 (64.5)	NA
Scalp	54 (17.5)	48 (23.2)	3 (4.3)	3 (9.7)	NA
Inverse	12 (3.9)	12 (5.8)	0	0	NA
Pustular	35 (11.4)	24 (11.6)	9 (12.9)	2 (6.5)	NA
Nail involvement	22 (7.1)	20 (9.7)	0	2 (6.5)	NA
Cutaneous and nail	44 (14.3)	24 (11.6)	16 (22.9)	4 (12.9)	NA
PASI	7.8 [4.2–11]	8.9 [5–12]	6.6 [4.1–10.9]	3.6 [1–6.6]	0.003
Psoriasis family history	115 (37.3)	77 (45.8)	27 (40.9)	11 (37.9)	NS
PsA family history	23 (7.5)	16 (10.7)	7 (11.7)	0	NS
Time (months) between onset of symptoms and diagnosis of psoriasis	6 [0–24]	6 [0.3–26.5]	3 [0–18.5]	16 [1.5–48.8]	NS
Comorbidities					
Smoker/former smoker	195 (64.1)	142 (69.6)	37 (52.9)	16 (53.3)	0.018
Alcohol consumption	19 (6.2)	17 (8.2)	1 (1.4)	1 (3.2)	NA
Overweight/obesity	112 (36.4)	95 (45.9)	14 (20)	3 (9.7)	<0.001
Hypertension	79 (25.6)	57 (27.5)	18 (25.7)	4 (12.9)	NS
Dyslipidemia	135 (43.8)	102 (49.3)	22 (31.4)	11 (35.5)	0.021
Diabetes	30 (9.7)	21 (10.1)	6 (8.6)	3 (9.7)	NS
Depression	23 (7.5)	18 (8.7)	4 (5.7)	1 (3.2)	NS
Hepatic steatosis	45 (14.6)	35 (16.9)	9 (12.9)	1 (3.2)	NS
COPD	12 (3.9)	7 (3.4)	3 (4.3)	2 (6.5)	NA
Hyperuricemia	26 (8.4)	19 (9.2)	5 (7.1)	2 (6.5)	NS
OSA	10 (3.2)	6 (2.9)	0	4 (12.9)	NA
Extracutaneous variables					
Musculoskeletal					
Symptoms	92 (29.9)	54 (26.1)	27 (38.6)	11 (35.5)	NS
Uveitis	2 (0.6)	1 (0.5)	1 (1.4)	0	NA
IBD	2 (0.6)	2 (1)	0	0	NA
CRP	2.4 (0.8–4.9)	3.1 (0.9–4.9)	2.2 (0.6–4.2)	4.1 (0.4–10)	NS
HLA B27-positive	13 (8.5)	12 (8.6%)	0	1 (25)	NA
Treatments					
TNF inhibitors	127 (41.2)	127 (61.4)	0	0	<0.001
Anti-IL17	38 (12.3)	38 (18.4)	0	0	<0.001
Anti-IL12/23	67 (21.8)	67 (32.4)	0	0	<0.001
Anti-IL23	48 (15.6)	48 (23.2)	0	0	<0.001
Apremilast	67 (21.8)	67 (32.4)	0	0	<0.001
Jak inhibitor	1 (0.3)	1 (0.5)	0	0	NA
Methotrexate	202 (65.6)	156 (75.4)	46 (65.7)	0	<0.001
Cyclosporine	103 (33.4)	85 (41.4)	18 (25.7)	0	<0.001
Acitretin	156 (50.6)	110 (53.1)	46 (65.7)	0	<0.001
Phototherapy	301 (97.7)	200 (96.6)	70 (100)	31 (100)	NA

Data are shown as n (%) and mean ± standard deviation or median [interquartile range p25–p75]. TREATMENTS: patients received treatment during the disease course and could have been taking any treatment previously. Group 1: receiving biologics or small molecules with or without csDMARDs. Group 2: receiving systemic conventional treatment (methotrexate, acitretin, or cyclosporine). Group 3: receiving non-pharmacological treatments such as phototherapy or topical agents. PsA: psoriatic arthritis; NA: not applicable; COPD: chronic obstructive pulmonary disease; OSA: obstructive sleep apnea; C-reactive protein (high-sensitivity-CRP values normal range: <5 mg/L; In the table results were expressed by median [IQR]; IBD: inflammatory bowel disease; NS: not significant; PASI: psoriasis activity and severity index.

**Table 2:** Demographic and clinical characteristics of psoriatic arthritis patients.

	n = 36 (%)
Demographic data	
Age	55.7 ± 15.53
Female sex	19 (52.8)
Variables related to psoriasis	
Type of psoriasis	
Plaque	10 (27.8)
Scalp	6 (16.7)
Inverse	3 (8.3)
Pustular	2 (5.5)
Nail involvement	1 (2.78)
Cutaneous and nail	14 (38.9)
PASI	8 + 4.9
Psoriasis family history	14 (38.9)
PsA family history	5 (13.9)
Time (months) between onset of symptoms and diagnosis of psoriasis	7 (0–12)
Variables related to PsA	
Type of PsA	
Peripheral	22 (66.1)
Axial	6 (16.7)
Peripheral and axial	8 (22.2)
Time (months) between onset of symptoms and diagnosis of psoriasis	11 [0–12]
Comorbidities	
Smoker/former smoker	20 (55.5)
Alcohol consumption	3 (8.3)
Overweight/obesity	14 (38.9)
Hypertension	12 (33)
Dyslipidemia	18 (50)
Diabetes	6 (16.7)
Depression	5 (13.9)
Hepatic steatosis	7 (19.4)
COPD	1 (2.8)
Hyperuricemia	3 (8.3)
OSA	5 (13.9)
Extracutaneous and extramusculoskeletal variables	
Uveitis	1 (2.8)
IBD	0
CRP	3.1 [0.7–11]
HLA B27-positive	6 (17.6)
Psoriasis treatment at diagnosis of PsA	
csDMARDs	16 (44.4)
Methotrexate	11 (30.5)
Cyclosporine	4 (11.1)
Acitretin	1 (2.7)
Biologics	11 (30.5)
TNF inhibitors	6 (16.6)
Anti-IL17	1 (2.7)
Anti-IL12/23	2 (5.5)
Anti-IL23	2 (5.5)
Treatments	
TNF inhibitors	23 (63.9)
Anti-IL17	7 (19.44)
Anti-IL12/23	12 (33.3)
Anti-IL23	11 (30.6)
Apremilast	11 (30.6)
Jak inhibitor	0
Methotrexate	30 (83.3)
Cyclosporine	10 (27.8)
Acitretin	17 (47.2)
Phototherapy	36 (100)
Number of biologics or small molecules (group 1)	
1	11 (34.4)
2	14 (43.7)
3	4 (12.5)
>3	3 (8.35)
Time (years) from psoriasis and PsA diagnosis	7.5 (2–20)
Group 1	7.5 (2–20)
Group 2	21 (4–22.5)
Group 3	2 [2–2]
Time (months) from musculoskeletal symptoms to PsA	15 [5.5–34.7]
Group 1	11 [4–34.5]
Group 2	25 [19.5–46]
Group 3	NA

Data are shown as n (%) and mean ± standard deviation or median [interquartile range p25–p75]. & TREATMENTS: patients received treatment during the disease course and could have been taking any treatment previously. Group 1: receiving biologics or small molecules with or without csDMARDs. Group 2: receiving systemic conventional treatment (methotrexate, acitretin, or cyclosporine). Group 3: receiving non-pharmacological treatments such as phototherapy or topical agents. PsA: psoriatic arthritis, NA: not applicable, COPD: chronic obstructive pulmonary disease, OSA: obstructive sleep apnea, CRP: C-reactive protein (high-sensitivity-CRP values normal range: <5 mg/L; In the table results were expressed by median [IQR], csDMARD: conventional synthetic disease-modifying antirheumatic drug; IBD: inflammatory bowel disease; PASI: psoriasis activity and severity index.

**Table 3:** Factors associated with psoriatic arthritis.

Variables	Univariate		Multivariate	
	OR (95% CI)	*p* Value	OR (95% CI)	*p* Value
Age at diagnosis	1 (0.9–1)	NS		
Female sex	1.1 (0.5–2.3)	NS		
Time (years) from onset of psoriasis symptoms and diagnosis of PsA	0.9 (0.9–1)	NS		
Time (years) from diagnosis of psoriasis until initiation of psoriasis treatment	1 (0.9–1)	NS		
Disease duration	0.9 (0.9–0.9)	0.043	0.9 (0.9–1)	0.051
PASI	0.9 (0.9–1)	NS		
Smoker/former smoker	0.6 (0.3–1.3)	NS		
Alcohol consumption	1.4 (0.4–5.2)	NS		
OSA	8.6 (2.3–31.4)	0.001	19.5 (3.5–108.9)	<0.001
Hepatic steatosis	1.4 (0.6–3.6)	NS		
Depression	2.2 (0.7–6.5)	NS		
Overweigh and obesity	1.1 (0.5–2.3)	NS		
Family history of psoriasis	0.9 (0.4–2.1)	NS		
Family history of PsA	0.4 (0.1–1.2)	NS		
Treatment group				
Group 1				
Group 2	0.2 (0.07–0.8)	0.023	0.262 (0.07–0.9)	0.034
Group 3	0.1 (0.02–1.3)	NS	0.067 (0.006–7.55)	0.029
Type of psoriasis				
Nail involvement	0.3 (0.04–2.6)	NS		
Nail only or nail and skin	3.09 (1.4–6.4)	0.002	3.605 (1.66–7.83)	0.001

NS: not significant; OSA: obstructive sleep apnea.

## Data Availability

The original contributions presented in the study are included in the article; further inquiries can be directed to the corresponding author.

## Abbreviations

The following abbreviations are used in this manuscript:

PsA	Psoriatic arthritis
csDMARD	Conventional synthetic disease-modifying anti-rheumatic drugs
OSA	Obstructive sleep apnea
IMID	immune-mediated inflammatory disease
PASI	Psoriasis Area and Severity Index
CRP	C-reactive protein
bDMARDs	Biologic disease-modifying anti-rheumatic drugs

## References

[1] Ferrándiz C., Carrascosa J.M., Toro M. (2014). Prevalence of psoriasis in Spain in the age of biologics. Actas Dermo-Sifiliogr..

[2] Gladman D.D., Antoni C., Mease P., Clegg D.O., Nash P. (2005). Psoriatic arthritis: Epidemiology, clinical features, course, and outcome. Ann. Rheum. Dis..

[3] Coates L.C., Helliwell P.S. (2017). Psoriatic arthritis: State of the art review. Clin. Med..

[4] Armstrong A.W., Harskamp C.T., Armstrong E.J. (2012). The association between psoriasis and obesity: A systematic review and meta-analysis of observational studies. Nutr. Diabetes.

[5] Armstrong E.J., Harskamp C.T., Armstrong A.W. (2013). Psoriasis and major adverse cardiovascular events: A systematic review and meta-analysis of observational studies. J. Am. Heart Assoc..

[6] Kurd S.K., Troxel A.B., Crits-Christoph P., Gelfand J.M. (2010). The risk of depression, anxiety, and suicidality in patients with psoriasis: A population-based cohort study. Arch. Dermatol..

[7] Liang S.E., Cohen J.M., Ho R.S. (2019). Psoriasis and suicidality: A review of the literature. Dermatol. Ther..

[8] Zabotti A., De Lucia O., Sakellariou G., Batticciotto A., Cincinelli G., Giovannini I., Idolazzi L., Maioli G., Tinazzi I., Aletaha D. (2021). Predictors, risk factors, and incidence rates of psoriatic arthritis development in psoriasis patients: A systematic literature review and meta-analysis. Rheumatol. Ther..

[9] Zabotti A., De Marco G., Gossec L., Baraliakos X., Aletaha D., Iagnocco A., Gisondi P., Balint P.V., Bertheussen H., Boehncke W.H. (2023). EULAR points to consider for the definition of clinical and imaging features suspicious for progression from psoriasis to psoriatic arthritis. Ann. Rheum. Dis..

[10] Gisondi P., Bellinato F., Maurelli M., Geat D., Zabotti A., McGonagle D., Girolomoni G. (2022). Reducing the risk of developing psoriatic arthritis in patients with psoriasis. Psoriasis.

[11] FitzGerald O., Haroon M., Giles J.T., Winchester R. (2015). Concepts of pathogenesis in psoriatic arthritis: Genotype determines clinical phenotype. Arthritis Res. Ther..

[12] Scher J.U., Ogdie A., Merola J.F., Ritchlin C. (2019). Preventing psoriatic arthritis: Focusing on patients with psoriasis at increased risk of transition. Nat. Rev. Rheumatol..

[13] Monteleone G., Moscardelli A., Colella A., Marafini I., Salvatori S. (2023). Immune-mediated inflammatory diseases: Common and different pathogenic and clinical features. Autoimmun. Rev..

[14] Sherlock J.P., Joyce-Shaikh B., Turner S.P., Chao C.C., Sathe M., Grein J., Gorman D.M., Bowman E.P., McClanahan T.K., Yearley J.H. (2012). IL-23 induces spondyloarthropathy by acting on RORγt^+^CD3^+^CD4^−^CD8^−^ entheseal resident T cells. Nat. Med..

[15] Yang K., Oak A.S.W., Elewski B.E. (2021). Use of IL-23 inhibitors for the treatment of plaque psoriasis and psoriatic arthritis: A comprehensive review. Am. J. Clin. Dermatol..

[16] Bellinato F., Chiricozzi A., Piaserico S., Targher G., Gisondi P. (2022). Could targeted pharmacotherapies exert a “disease modification effect” in patients with chronic plaque psoriasis?. Int. J. Mol. Sci..

[17] Gisondi P., Bellinato F., Targher G., Idolazzi L., Girolomoni G. (2022). Biological disease-modifying antirheumatic drugs may mitigate the risk of psoriatic arthritis in patients with chronic plaque psoriasis. Ann. Rheum. Dis..

[18] Rosenthal Y.S., Schwartz N., Sagy I., Pavlovsky L. (2022). Incidence of psoriatic arthritis among patients receiving biologic treatments for psoriasis: A nested case-control study. Arthritis Rheumatol..

[19] Haberman R.H., MacFarlane K.A., Catron S., Samuels J., Blank R.B., Toprover M., Uddin Z., Hu J., Castillo R., Gong C. (2022). Efficacy of guselkumab, a selective IL-23 inhibitor, in preventing arthritis in a multicentre psoriasis at-risk cohort (PAMPA): Protocol of a randomized, double-blind, placebo-controlled multicentre trial. BMJ Open.

[20] Taylor W., Gladman D., Helliwell P., Marchesoni A., Mease P., Mielants H. (2006). CASPAR Study Group. Classification criteria for psoriatic arthritis: Development of new criteria from a large international study. Arthritis Rheum..

[21] Fredriksson T., Pettersson U. (1978). Severe psoriasis—Oral therapy with a new retinoid. Dermatology.

[22] Alinaghi F., Calov M., Kristensen L.E., Gladman D.D., Coates L.C., Jullien D., Gottlieb A.B., Gisondi P., Wu J.J., Thyssen J.P. (2019). Prevalence of psoriatic arthritis in patients with psoriasis: A systematic review and meta-analysis of observational and clinical studies. J. Am. Acad. Dermatol..

[23] World Medical Association (2013). World Medical Association Declaration of Helsinki: Ethical principles for medical research involving human subjects. J. Am. Med. Assoc..

[24] Kang Z., Zhang X., Du Y., Dai S.M. (2024). Global and regional epidemiology of psoriatic arthritis in patients with psoriasis: A comprehensive systematic analysis and modelling study. J. Autoimmun..

[25] Floris A., Mugheddu C., Sichi L., Anedda J., Frau A., Sorgia J., Li Volsi L., Paladino M.T., Congia M., Chessa E. (2025). Treatment of psoriasis with different classes of biologics reduces the likelihood of peripheral and axial psoriatic arthritis development. Rheumatology.

[26] López Estebaranz J.L., Zarco-Montejo P., Samaniego M.L., García-Calvo C. (2015). Prevalence and clinical features of psoriatic arthritis in psoriasis patients in Spain. Eur. J. Dermatol..

[27] Carrascosa J.M., Puig L., Belinchón Romero I., Salgado-Boquete L., Del Alcázar E., Andrés Lencina J.J., Moreno D., de la Cueva P. (2022). Actualización práctica de las recomendaciones del Grupo de Psoriasis de la Academia Española de Dermatología y Venereología (GPS) para el tratamiento de la psoriasis con terapia biológica. Parte 1. «Conceptos y manejo general de la psoriasis con terapia biológica». Actas Dermo-Sifiliogr..

[28] Nast A., Smith C., Spuls P.I., Avila Valle G., Bata-Csörgo Z., Boonen H., De Jong E., Garcia-Doval I., Gisondi P., Kaur-Knudsen S. (2021). EuroGuiDerm Guideline on the systemic treatment of Psoriasis vulgaris—Part 1: Treatment and monitoring recommendations. Br. J. Dermatol..

[29] Watad A., Zabotti A., Patt Y.S., Gendelman O., Dotan A., Ben-Shabat N., Fisher L., McGonagle D., Amital H. (2024). From Psoriasis to Psoriatic Arthritis: Decoding the Impact of Treatment Modalities on the Prevention of Psoriatic Arthritis. Rheumatol. Ther..

[30] Felquer M.L.A., LoGiudice L., Galimberti M.L., Rosa J., Mazzuoccolo L., Soriano E.R. (2022). Treating the skin with biologics in patients with psoriasis decreases the incidence of psoriatic arthritis. Ann. Rheum. Dis..

[31] Meer E., Merola J.F., Fitzsimmons R., Love T.J., Wang S., Shin D., Chen Y., Xie S., Choi H., Zhang Y. (2022). Does biologic therapy impact the development of PsA among patients with psoriasis?. Ann. Rheum. Dis..

[32] Cohen J.M., Jackson C.L., Li T.Y., Wu S., Qureshi A.A. (2015). Sleep disordered breathing and the risk of psoriasis among US women. Arch. Dermatol. Res..

[33] Yang Y.W., Kang J.H., Lin H.C. (2012). Increased risk of psoriasis following obstructive sleep apnea: A longitudinal population-based study. Sleep Med..

[34] Ger T.Y., Fu Y., Chi C.C. (2020). Bidirectional Association Between Psoriasis and Obstructive Sleep Apnea: A Systematic Review and Meta-Analysis. Sci. Rep..

